# Steroid-Induced Osteonecrosis of the Femoral Head: Novel Insight Into the Roles of Bone Endothelial Cells in Pathogenesis and Treatment

**DOI:** 10.3389/fcell.2021.777697

**Published:** 2021-11-30

**Authors:** Cheng Huang, Zeqin Wen, Junjie Niu, Subin Lin, Weiguo Wang

**Affiliations:** ^1^ Department of Orthopedics, China-Japan Friendship Hospital, Beijing, China; ^2^ Department of Orthopedics, Xiangya Hospital, Central South University, Changsha, China; ^3^ Department of Orthopedics, The First Affiliated Hospital of Soochow University, Suzhou, China; ^4^ Department of Orthopedics, The Second Affiliated Hospital of Soochow University, Suzhou, China

**Keywords:** steroid-induced osteonecrosis of the femoral head, bone endothelial cells, bone microvascular endothelial cells, bone progenitor cells, angiogenesis, pathogenesis, treatment

## Abstract

Steroid-induced osteonecrosis of the femoral head (SONFH) is a disease characterized by the collapse of the femoral head. SONFH occurs due to the overuse of glucocorticoids (GCs) in patients with immune-related diseases. Among various pathogenesis proposed, the mechanism related to impaired blood vessels is gradually becoming the most convincing hypothesis. Bone endothelial cells including bone microvascular endothelial cells (BMECs) and endothelial progenitor cells (EPCs) play a crucial role in the maintenance of vascular homeostasis. Therefore, bone endothelial cells are key regulators in the occurrence and progression of SONFH. Impaired angiogenesis, abnormal apoptosis, thrombosis and fat embolism caused by the dysfunctions of bone endothelial cells are considered to be the pathogenesis of SONFH. In addition, even with high disability rates, SONFH lacks effective therapeutic approach. Icariin (ICA, a flavonoid extracted from Epimedii Herba), pravastatin, and VO-OHpic (a potent inhibitor of PTEN) are candidate reagents to prevent and treat SONFH through improving above pathological processes. However, these reagents are still in the preclinical stage and will not be widely used temporarily. In this case, bone tissue engineering represented by co-transplantation of bone endothelial cells and bone marrow mesenchymal stem cells (BMSCs) may be another feasible therapeutic strategy.

## Introduction

Glucocorticoids have been widely used in the treatment of rheumatic diseases, autoimmune diseases and allergic diseases (Yao et al., 2020). However, long-term or extensive GCs use may lead to steroid-induced osteonecrosis of the femoral head (SONFH). SONFH is a disabling orthopedic disease, which is characterized by the progressive deterioration of the hip joint in individuals aged 20–50 years old (Kong et al., 2020; Yu et al., 2020). Although many pathophysiological mechanisms for SONFH have been proposed, such as impaired microcirculation, imbalance between osteogenic and adipogenic differentiation, fat embolism, coagulation disorder and intramedullary pressure change, significant gaps remain in the understanding of the pathogenesis of SONFH (Li et al., 2005; Murata et al., 2007; Yeh et al., 2008; Yue et al., 2021). Among several existing mechanisms for SONFH, the vascular hypothesis seems to be the most convincing and influential (Kerachian et al., 2006).

As long ago as 1935, Phemister raised that vascular impairment led to thrombosis and embolism contributing to the progression of avascular necrosis of the femoral head (ANFH) (Phemister, 1935). It was not until Hirano et al. that direct histological evidence, severe luminal stenosis of the draining vein in the early stages of ANFH, was observed (Hirano et al., 1997). In another study, Starklint et al. have found a wide obstruction of vessels in the late stages of ANFH and the venous outflow is further damaged by thrombus and perivenous concentric fibrosis, which immensely reduces the lumen of veins (Starklint et al., 1995). In addition, osteocytes cannot survive more than 100 mm from blood vessels, so it is widely believed that vascular development always precedes osteogenesis (Yu et al., 2009). However, with the deepening understanding of bone formation and repair, “angiogenic-osteogenic coupling” concept has been established (Riddle et al., 2009). SONFH is a type of ANFH. As a result, blood vessels play a key role in the pathogenesis and repair of SONFH. Currently, vascular hypothesis assumes that GCs could reduce the number of blood vessels, decrease the regional blood flow of femoral head and lead to SONFH (Kerachian et al., 2006; Kerachian et al., 2009a).

Bone endothelial cells mainly refer to bone microvascular endothelial cells (BMECs) and endothelial progenitor cells (EPCs) that can differentiate into BMECs. BMECs line the sinusoids and inner layer of blood vessels, which play a crucial role in vascular homeostasis and angiogenesis (Kusumbe et al., 2014). It is reported that femoral head microcirculation disorder secondary to BMECs dysfunction is of great significance in the occurrence and progression of SONFH (Nishimura et al., 1997; Kerachian et al., 2009b). Similarly, as the precursor cells of BMECs, EPCs are involved in maintaining the physiological structure and function of vascular endothelium (Yao et al., 2020). Several studies have shown that the number and function of circulating EPCs in patients with SONFH are impaired (Feng et al., 2010; Chen et al., 2013; Ding P. et al., 2019). Given to the importance of vascular hypothesis in SONFH, researches about the effects of BMECs and EPCs on the blood supply of femoral head are helpful to further understand the pathogenesis of SONFH.

Moreover, drug treatment (e.g., anticoagulants, fibrinolysis-enhancing drugs, blood vessel dilatators and lipid-reducing drugs) combined with hip-preserving surgery (e.g., core decompression, bone transplantation and osteotomy) can be applied to treat early ONFH (Zhao et al., 2020). However, these treatments are less effective, as more than 80% of patients with ONFH eventually require total hip arthroplasty (THA) (Johnson et al., 2014). Although THA significantly improves the living quality of patients, it cannot be considered as the best therapy for ONFH because of dislocation, periprosthetic fracture, infection and prosthesis loosening after THA, especially in relatively young patients (Xu et al., 2021). Therapeutic strategies designed according to the pathophysiological role of BMECs and EPCs in SONFH pathogenesis may be effective.

In this review, we summarize the novel roles of bone endothelial cells in the pathogenesis and treatment of SONFH. Impaired angiogenesis, abnormal apoptosis, thrombosis and fat embolism caused by the dysfunctions of bone endothelial cells are considered to be the pathogenesis of SONFH. Targeting to repair the amount and function of bone endothelial cells or co-transplantation of bone endothelial cells and bone marrow mesenchymal stem cells (BMSCs) may be effective therapeutic approaches with great application potential. Furthermore, it is promising to point out the direction of future studies on the pathogenesis and treatment of SONFH.

## Bone Microvascular Endothelial Cells

As previously mentioned, BMECs line the interior surface of bone microvessels and sinuses, maintaining local blood supply in femoral head. Besides, the reduction of blood flow in femoral head plays a vital role in the pathogenesis of ANFH (Kerachian et al., 2006). Therefore, BMECs damage may be the critical factor to promote the progression of SONFH.

Recent studies have demonstrated the existence of two types of BMECs: type H and type L endothelial cells (Kusumbe et al., 2014). Type H BMECs are mainly located in metaphysis and highly express CD31 and endomucin (EMCN), while type L BMECs are mainly located in the diaphysis and lowly express CD31 and EMCN (Zhang J. et al., 2020). Runx2^+^ osteoprogenitors and collagen type 1α^+^ osteoblasts are abundant around the type H BMECs in the metaphysis and endosteum, suggesting type H BMECs could promote bone repair and regeneration (Kusumbe et al., 2014). However, there is almost no osteoprogenitor surrounding type L BMECs (Kusumbe et al., 2014; Xu et al., 2018). At present, there are few studies on the role of type H BMECs in the pathogenesis of SONFH. Some studies have even shown contradictory results, which may be attributed to the differences in preclinical animal models (Zhou et al., 2017; Lane et al., 2018; Peng et al., 2020). And whether targeting type H BMECs can reverse the pathological processes of SONFH remains unclear. Hence this review mainly focuses on recent research progresses of BMECs in SONFH.

### Animal Experiments of Bone Microvascular Endothelial Cells

Patients with SONFH have common pathological features of allergic vasculitis prior to hormone administration (Saito et al., 1992). Lipopolysaccharide (LPS) stimulates the immune system and induces the pathological changes of the blood system. Therefore, the combination of LPS and methylprednisolone (MPS) to induce SONFH in Sprague-Dawley (SD) rats is consistent with human clinical pathological features (Saito et al., 1992). At the same time, femoral tissues of SD rats are collected for pathological examination to determine whether SONFH models are successfully established (Drescher et al., 2011). And the BMECs used *in vitro* were isolated from the femoral head of SONFH rat models. Animal experiments including *in vivo* and *in vitro* SONFH models were established using the above methods.

So far, mechanisms regarding how the glucocorticoid takes effect on BMECs in animal experiments mainly focused on MicroRNAs (miRNAs). miRNAs are a group of small 18–25-nt-long non-coding RNAs (Krol et al., 2010). They are involved in plenty of physiological and pathological processes by modulating the transcription or post-transcriptional translation to silence the expression of their target genes (Ambros, 2004; Lan et al., 2015). Four miRNAs differentially expressed in BMECs of SONFH rats were identified by real-time quantitative polymerase chain reaction (qPCR) and gene microarray, including two up-regulated (miR-335, miR-132-3p) and two down-regulated (miR-466b-2-3p, let-7c-1-3p) (Yue et al., 2018). Moreover, Yue et al. reported that miR-335 could down-regulate the expression of endothelial nitric oxide synthase (eNOS), superoxide dismutase 2 (SOD2) and Ras p21 protein activator 1 (RASA1) (Yue et al., 2018). eNOS is a specific protease in BMECs, which has a variety of physiological effects, such as vasodilation, anti-platelet aggregation, and promoting functional repair of impaired BMECs (Yue et al., 2018). SOD is an antioxidant enzyme that catalyzes the reactive oxygen species (ROS) into hydrogen peroxide and oxygen molecules to inhibit senescence and apoptosis (Nguyen et al., 2020). RASA1 is a modulator of Ras GDP and GTP and plays an important role in several physiological processes such as angiogenesis, cell proliferation and apoptosis (Zhang Y. et al., 2020). In addition, Lei et al. observed that miR-132-3p expression was significantly up-regulated after femoral artery occlusion, and the hind limb perfusion recovery after ischemia was slower in knockout mice compared with wild-type mice (Lei et al., 2015; Yue et al., 2018). Therefore, miR-335 and miR-132-3p may be involved in regulating the functional repair of impaired BMECs and angiogenesis in SONFH. However, the effects of rno-let-7c-1-3p and rno-miR-466b2-3p on proliferation and apoptosis of BMECs have not been reported (Yue et al., 2018). In the meantime, no studies have evaluated the role of glucocorticoid receptor (GR) on BMECs in the pathogenesis of SONFH. Whereas, a recent study investigated GR on mouse endothelial cells, identifying the pivotal role of Wnt signaling pathway in suppressing vascular inflammation *via* GR (Zhou et al., 2020). This result may guide the further research of signaling pathways mediated by GR on BMECs, which function as key factors in SONFH pathogenesis.

As a flavonoid extracted from Epimedii Herba, Icariin (ICA) has been widely used to promote bone healing, improve osteoporosis and SONFH in China, Japan and Korea (Brandi and Collin-Osdoby, 2006; Zhang et al., 2007; Zhu et al., 2012; Sun et al., 2015). Yue et al. (2021) observed that though still higher than the control group, the expression of miR-335 was markedly decreased after ICA treatment *in vivo*. What’s more, they also found ICA had a modulatory effect on 101 unconventionally expressed target genes of miR-335 (Yue et al., 2021). As a result, down-regulating the expression of miR-335 may be the mechanism of ICA to prevent and therapy SONFH. In addition, Wen et al. (2008a) observed the increased ratio of empty lacunae, the sparse capillary network, and the partially blocked blood vessels in necrotic femoral head tissue from SONFH rabbits. However, ICA treatment can significantly decrease the rate of empty lacunae and relatively up-regulate the expression of angiogenic biomarker CD31 *in vivo* (Yu et al., 2019). And the tube formation and osteogenesis-related cytokines expression of BMECs can be stimulated by ICA *in vitro* (Yu et al., 2019). These results both *in vitro* and *in vivo* suggest that ICA may be a potential drug in the treatment of SONFH. However, rat models are far too different from human beings to infer similar therapeutic roles in humans.

### Human Experiments of Bone Microvascular Endothelial Cells

There are two ways to establish the BMECs model with SONFH used in human experiments (Lu et al., 2020; Yu et al., 2020). One is to isolate BMECs from patients with SONFH and indications for THA, the other is to extract BMECs from patients with femoral neck fractures who have undergone THA. Yu et al. (2020) demonstrated that BMECs from SONFH patients had down-regulated angiogenic abilities. Endothelial function has been reported to decline with an increasing age in healthy individuals (Yavuz et al., 2008). However, dysfunction of BMECs was observed even when the mean age of the control group was significantly older than that of the SONFH group (Yu et al., 2020). This fully confirms that GCs can promote the progression of the dysfunction of BMECs from SONFH patients. However, the research results might not be replicated in the local microenvironment of the femoral head in the presence of SONFH because the study was conducted *in vitro* (Yu et al., 2020).

Similarly, Yu et al. (2019) also reported that hydrocortisone significantly inhibited the expression of angiogenic cytokines and the activation of Akt in BMECs, which decreased the migration and tube formation activities of BMECs. Angiogenic cytokines including vascular endothelial growth factor (VEGF), CD31, von Willebrand factor (vWF) and platelet-derived growth factor-B (PDGF-B) are promotors or markers of angiogenesis mainly expressed in BMECs (Yang et al., 2003; Muraoka et al., 2005; Uras et al., 2012; Mittermayr et al., 2016). It has been reported that the activation of survival signal PI3K/Akt pathway is related to angiogenesis (Lee et al., 2014). Since blood supply is critical to the maintenance of femoral head structure and function, dysfunction of BMECs and inhibited angiogenesis are potential mechanisms for SONFH (Kerachian et al., 2006).

Besides, some studies have found that GCs-induced apoptosis of BMECs can activate thrombosis and decrease angiogenesis, secondary by infarction and ischemia (Vogt and Schmid-Schönbein, 2001; Kerachian et al., 2006). B cell lymphoma-2 (Bcl-2), as an oncoprotein, has a significant effect on inhibiting apoptosis, while Bcl-2 associated X (Bax) has an obvious effect on promoting apoptosis (Nomura et al., 1999; Delbridge et al., 2016). Therefore, it’s the balance between Bcl-2 and Bax that determines apoptosis. Furthermore, caspase-3 is a key factor in the activation of apoptosis (Porter and Jänicke, 1999). Yu et al. found the expression of Bcl-2 was significantly down-regulated, while the expression of Bax and cleaved caspase-3 were increased in BMECs with SONFH (Yu et al., 2019; Yu et al., 2020). These results demonstrate that the apoptosis of BMECs functions a lot in the progression of SONFH.

In addition to impaired angiogenesis and increased apoptosis of BMECs, Li et al. (2004) reported that the hypercoagulability and hypofibrinolysis state induced by dysfunction of BMECs may be the pathological mechanism of SONFH as well. eNOS and endothelin 1 (ET-1) are two vasoactive factors with opposite functions secreted by BMECs, whose balance plays an important role in regulating vasomotor (Lu et al., 2020). ET-1 is the strongest vasoconstrictor until now and acts by binding to receptors on BMECs and vascular smooth muscle cells, while eNOS is a vasodilator and anticoagulation that acts by inhibiting the secretion and function of ET-1, platelet aggregation and intercellular adhesion (Houde et al., 2016; Hong et al., 2019). Angiotensin II (Ang II) binds to receptors on BMECs to inhibit eNOS expression and damage BMECs (Shatanawi et al., 2015). Prostaglandin I_2_ (PGI_2_) is secreted by BMECs and significantly expands blood vessels and suppresses platelet aggregation by activating prostacyclin receptors (IP receptors) in BMECs and platelets (Shatanawi et al., 2015). Prostaglandin E (PGE) is capable of expanding blood vessels, protecting BMECs and increasing the expression of eNOS (Fang et al., 2010). Plasminogen activator inhibitor-1 (PAI-1) is the inhibitor of tissue plasminogen activator (t-PA) primarily produced by BMECs, the increased expression of which can promote intravascular thrombosis (Ghosh and Vaughan, 2012). Intercellular adhesion molecule 1 (ICAM-1), an important adhesion molecule, mediates adhesion between leukocytes, inflammatory cells and BMECs (Bui et al., 2020). Lu et al. found that the expressions of ET-1 receptor, Ang II receptor and ICAM-1 were dramatically increased and the expressions of eNOS, PGI_2_ synthase, PGE synthase, PGE receptor and VEGF were dramatically decreased after 24-h GCs treatment (Lu et al., 2020). However, the expression of ET-1 was dramatically down-regulated, suggesting that the effect of GCs on BMECs is complex and needs further investigations. In other words, vasoconstriction and thrombosis were promoted after GCs-induced BMECs damage.

Yu et al. (2019) reported that ICA could promote angiogenesis by up-regulating the expression of CD31, vWF, PDGF-B in BMECs and activating Akt and reduce the apoptosis of BMECs by up-regulating Bax and down-regulating the expression of Bcl-2. Circular RNAs (circRNAs), serve as endogenous RNAs competing for miRNA binding sites, are regarded as new modulators of diseases (Wu et al., 2019). Mao et al. (2021) reported that CircCDR1as, functioning as a sponge for miR-135b/factor inhibiting hypoxia inducible factor 1 (FIH-1), reduced the expression of hypoxia inducible factor-1α (HIF-1α) and VEGF, and thereby inhibited the angiogenesis of BMECs. Research results above suggest that the administration of ICA or targeting to inhibit CircCDR1 as may be effective therapeutic strategies for SONFH. However, the therapeutic approaches are still in the pre-clinical stage and lack the support of clinical trials. In addition, there is a short of therapeutic strategies targeting thrombosis caused by BMECs damage. Therefore, further investigations are needed in the future in regard to the thrombosis of SONFH.

## Endothelial Progenitor Cells

EPCs are considered to be critical participants in endogenous vascular repair and regeneration by differentiating into mature endothelial cells (Kim et al., 2010; Balistreri et al., 2015). EPCs are primarily derived from bone marrow (Asahara et al., 2011). In addition, a small amount of EPCs are also found in umbilical cord blood, circulating blood and arterial walls (Doyle and Caplice, 2005; Wu et al., 2005; Finney et al., 2006; Pacilli and Pasquinelli, 2009). According to the difference in culture time, EPCs can be divided into two subgroups: early EPCs (eEPCs) and late EPCs (lEPCs) (Patel et al., 2016). In terms of maturation time, eEPCs appeared 4–7 days after culture, while lEPCs appeared 14–21 days after culture (Yang et al., 2018). eEPCs are characterized by several surface markers of progenitor cells, including CD14, CD31, CD34, CD45, CD133 and vWF (Recchioni et al., 2016). eEPCs have a weak proliferation capacity, but can secrete a variety of cytokines, such as VEGF, hepatocyte growth factor (HGF), granulocyte colony-stimulating factor (G-CSF), and interleukin-8 (IL-8) (Rehman et al., 2003). However, lEPCs express endothelial markers such as KDR, VE-cadherin and CD146 with a strong capacity of proliferation and differentiation (Hirschi et al., 2008; Madonna and De Caterina, 2015). In fact, the antigen expression profile on the surface of EPCs remains controversial (Werner and Nickenig, 2006; Chen et al., 2013). When different combinations of surface antigens are selected, there may be some differences in experimental results (Ding P. et al., 2019).

EPCs have the potential to differentiate into any kinds of capillary endothelial cells, including BMECs (Peters, 2018). In addition, EPCs can be isolated noninvasively from the donors’ own peripheral blood and umbilical cord blood, as well as from human induced pluripotent stem cells (hiPSCs) to avoid immunogenicity problems (Boyer et al., 2000; Ingram et al., 2004; Mead et al., 2008). EPCs-differentiated endothelial *in vitro* and *in vivo* have similar permeability to vessel-derived endothelial, and are superior to vessel-derived endothelial in vascular network formation (Peters, 2018). Therefore, EPCs transplantation to promote angiogenesis at the lesion region has great prospects. One of the most important pathogenesis of SONFH is the suppression of angiogenesis caused by dysfunction of BMECs, so most of the previous studies on SONFH focused on the changes of BMECs. However, recent studies have found that EPCs are more involved in vascular repair and regeneration than BMECs, which makes EPCs the focus of interest in the pathogenesis and treatment of SONFH (Ding S. et al., 2019).

### Animal Experiments of Endothelial Progenitor Cells

Animal models for EPCs-related experiments were established by rats or rabbits treated with LPS and MPS/dexamethasone (Dex). Reduced blood flow and impaired blood supply to the femoral head caused by SONFH can lead to increased lactic acid levels resulting in an acidic local microenvironment (Song et al., 2010). Ovarian cancer G-protein-coupled Receptor 1 (OGR1) is a key receptor involved in sensing proton. Ding S. et al. (2019) found that OGR1 inhibited the proliferation, migration and angiogenesis of EPCs induced by acidic environment in SONFH. It means OGR1 may be a new breakthrough in treating SONFH. Moreover, it is well-known that stromal cell-derived factor-1α (SDF-1α), the product of CXCL12, promotes angiogenesis through the CXCL12/CXCR4 or CXCL12/CXCR7 signaling pathway (Dimova et al., 2019; Zhang et al., 2019). Kong et al. (2020) demonstrated that transplantation of miR-137-3p-silenced BMSCs can promote angiogenesis by up-regulating CXCL12/SDF-1α to mobilize EPCs into circulation. However, whether CXCL12/CXCR4 or CXCL12/CXCR7 signaling pathways is involved in the mobilization of EPCs remains unknown.

In addition to the impaired angiogenesis caused by the damage of EPCs, the apoptosis of EPCs is also one of the possible pathogenesises of SONFH. Liao et al. (2017) reported that suppressed mammalian target of rapamycin (mTOR) signal induced by the activations of glucocorticoid receptors down-regulates the HIF pathway and induces EPCs apoptosis, which may be the pathophysiological mechanism of SONFH. Meanwhile, there may be certain therapeutic potential in enhancing mTOR signal. Autophagy is a complex process in which cells adapt to degrade and recycle intracellular components under stress conditions, thus promoting cell survival (Hamacher-Brady et al., 2006; Eisenberg-Lerner et al., 2009). It was observed that autophagy increased in EPCs treated with Dex, but this change gradually attenuated with the prolongation of Dex treatment (Liao et al., 2018). At the same time, prolonged Dex treatment reduced cell viability, indicating that autophagy is beneficial for EPCs to respond to Dex stimulation and avoid damage (Liao et al., 2018). Liao et al. also reported that pravastatin activated AMP-activated protein kinase (AMPK) mediated by liver kinase B1 (LKB1), thereby inhibiting the mTOR signaling pathway, recovering autophagy of EPCs and protecting them from Dex-induced apoptosis (Liao et al., 2018). The above studies on mTOR signaling pathway have produced opposite conclusions, so the mechanism of mTOR signaling pathway in apoptosis of EPCs remains to be explored.

The extrinsic death receptor pathway and the intrinsic mitochondrial pathway are two main systems that initiate apoptosis (Thorburn, 2004). Phosphatase and tensin homolog (PTEN), a tumor-suppressor gene that enhances apoptosis, has recently been observed to be significantly elevated in the serum of patients with SONFH (Kotelevets et al., 2018; Li et al., 2018; Li et al., 2019). Moreover, Yao et al. (2020) found that GCs can induce EPCs apoptosis by activating mitochondrial pathway. VO-OHpic, a potent inhibitor of PTEN, could protect EPCs from apoptosis through inhibiting mitochondrial pathway (Yao et al., 2020). They also observed that GCs exposure resulted in mitochondrial fission and conspicuous abnormalities of ROS production and mitochondrial membrane potential (MMP), which promote the apoptosis of EPCs (Yao et al., 2020). Similarly, VO-OHpic could reverse these changes and protect EPCs. In addition, nuclear factor erythroid 2-related factor 2 (Nrf2) regulates the production of several antioxidant enzymes (Tavakkoli et al., 2019). VO-OHpic promotes angiogenesis and suppresses apoptosis of EPCs by activating Nrf2 (Yao et al., 2020). Therefore, VO-OHpic may be an effective strategy for the prevention and therapy of SONFH.

Bone tissue engineering is getting increasingly attractive to researchers because of the enormous potential for osteogenesis and angiogenesis. To enhance bone regeneration and angiogenesis at SONFH lesions, transplantation of BMSCs, EPCs and co-transplantation of both have been reported so far. BMSCs are considered to be ideal seed cells for SONFH treatment due to the enormous potential for self-renewal and multilineage differentiation, including osteogenesis and angiogenesis (Chen et al., 2019). However, several studies have reported that BMSCs isolated from proximal femur and iliac crest in SONFH patients have decreased osteogenic differentiation ability, limiting the application of BMSCs transplantation in SONFH treatment (Hernigou et al., 1999; Houdek et al., 2016). Although researchers have used gene transfection and established sustainable-release growth factor biomaterials to enhance BMSCs’ osteogenic and angiogenic abilities, the harm of gene transfection to human body and the construction of suitable biomaterials remain incomplete problems (Wen et al., 2008b; Wen et al., 2012; Amsden, 2015; Shapiro et al., 2018; Kong et al., 2019). For EPCs, they are not directly involved in osteogenesis because they cannot differentiate into osteoblasts (Xu et al., 2021). Both carboxymethyl chitosan (CMC) and alginate (ALG) possess outstanding biocompatibility in enhancing osteogenesis (Upadhyaya et al., 2013; Jain and Bar-Shalom, 2014). The composite scaffold can not only transport stem cells, but also provide a beneficial microenvironment for cell proliferation and intercellular communications (Xu et al., 2021). Therefore, CMC/ALG/BMSC/EPC composite scaffold have been developed for SONFH treatment and prevention.

Co-cultured BMECs and EPCs interact with each other through paracrine and direct intercellular contact to promote osteogenesis and angiogenesis has been verified as the main mechanism. Xu et al. demonstrated that BMSCs and EPCs mutually promote osteogenesis and angiogenesis through the secretion of various growth factors, such as VEGF and PDGF (Xu et al., 2021). Moreover, direct contact between EPCs and BMSCs can lead to endothelial-like phenotypic differentiation of BMSCs (Joddar et al., 2018). Implanted cells can promote tissue regeneration through proliferation, differentiation and paracrine (García-Sánchez et al., 2019). In addition to impaired osteogenesis and angiogenesis, lipid metabolism disturbance is another key promotor contributing to SONFH (Zhang et al., 2018). The imbalance between osteogenic and adipogenic differentiation of BMSCs may lead to adipocyte hypertrophy and fat embolism, reducing blood supply to the femoral head (Fukui et al., 2006). Transcription factors play a critical role in determining the fate of BMSCs. For instance, Runx2 and BMP-2 are crucial transcription factors that promote osteogenic differentiation of BMSCs, while PPARγ and C/EBPα are pivotal transcription factors that enhance adipogenic differentiation of BMSCs (Xu et al., 2021). The expression of Runx2 and BMP-2 was up-regulated in the co-cultured cells, while the expression of PPARγ and C/EBPα was down-regulated, resulting in BMSCs tending to differentiate into osteoblasts (Xu et al., 2021). However, the optimal ratio between BMSCs and EPCs in a co-transplantation system has yet to be determined. The SONFH lesion is in a state of hypoxia due to blood supply disorder, and the ability of proliferation, differentiation and cytokine secretion of co-cultured cells under hypoxia circumstances remains to be studied. In conclusion, BMSCs and EPCs co-transplantation is a promising therapeutic approach for SONFH.

### Human Experiments of Endothelial Progenitor Cells

Endothelial Progenitor Cells used in human experiments were isolated and extracted from patients with SONFH. Feng et al. observed a decrease in the number and function of circulating EPCs in patients with SONFH, such as suppressed migration, impaired angiogenesis, and increased senescence (Feng et al., 2010). Similarly, Ding P. et al. (2019) reported that low doses of GCs significantly inhibited angiogenesis of EPCs, while only large doses of GCs could significantly inhibited cell proliferation. Clinical routine doses of GCs may never reach the threshold of serum concentration that inhibit EPCs proliferation (Rouster-Stevens et al., 2008). And the decreased number of EPCs in patients with SONFH may as a result of the indirect effects of long-term exposure to GCs (Ding P. et al., 2019). In addition, GCs can down-regulate the expression of CXCR7 in EPCs and inhibit the downstream Akt and GSK-3β/Fyn signaling pathways of SDF-1/CXCR7 (Ding P. et al., 2019). Akt and GSK-3β/Fyn are involved in the angiogenesis of EPCs, and the up-regulation of Fyn caused by the decreased phosphorylation of GSK-3β can promote the degradation of Nrf2 (Torossian et al., 2014; Chen et al., 2015; Dai et al., 2017).

In addition, Chen et al. (2013) found that the migration and secretion of eEPCs were inhibited, while the proliferation and angiogenesis of lEPCs were significantly suppressed, which was appropriate for their different physiological functions. At the same time, the number of eEPCs and lEPCs were lower than that of the control group with similar conditions. Therefore, lEPCs may be a superior graft for SONFH compared to EPCs. Recent studies have reported successful enrichment and cultivation of lEPCs on a large scale, which greatly expanded the application prospect of lEPCs in bone tissue engineering (Reinisch et al., 2009; Kolbe et al., 2010).

In injured tissue, cells expressing CXCR4 are recruited through SDF-1 secreted by surrounding cells to promote healing of the injury (Ding and Tredget, 2015). Carolina et al. (2018) reported that GCs inhibited the migration and homing of umbilical cord blood (UCB) derived human EPCs to injury by down-regulating CXCR4 expression in both normoxic and hypoxic conditions. In normoxic conditions, GCs down-regulate CXCR4 expression in EPCs by damaging prostaglandin E2 (PGE2) synthases cyclooxygenase (COX2) and microsomal PGE2 synthase 1 (mPEGS1) and prostaglandin receptor EP4. While in hypoxic conditions, GCs down-regulate CXCR4 expression in EPCs through both PGE2 pathway and HIF2α pathway. However, whether GCs could influence the migration and homing ability of bone marrow derived EPCs in SONFH patients remains to be further investigated.

## Conclusion

**FIGURE 1:**
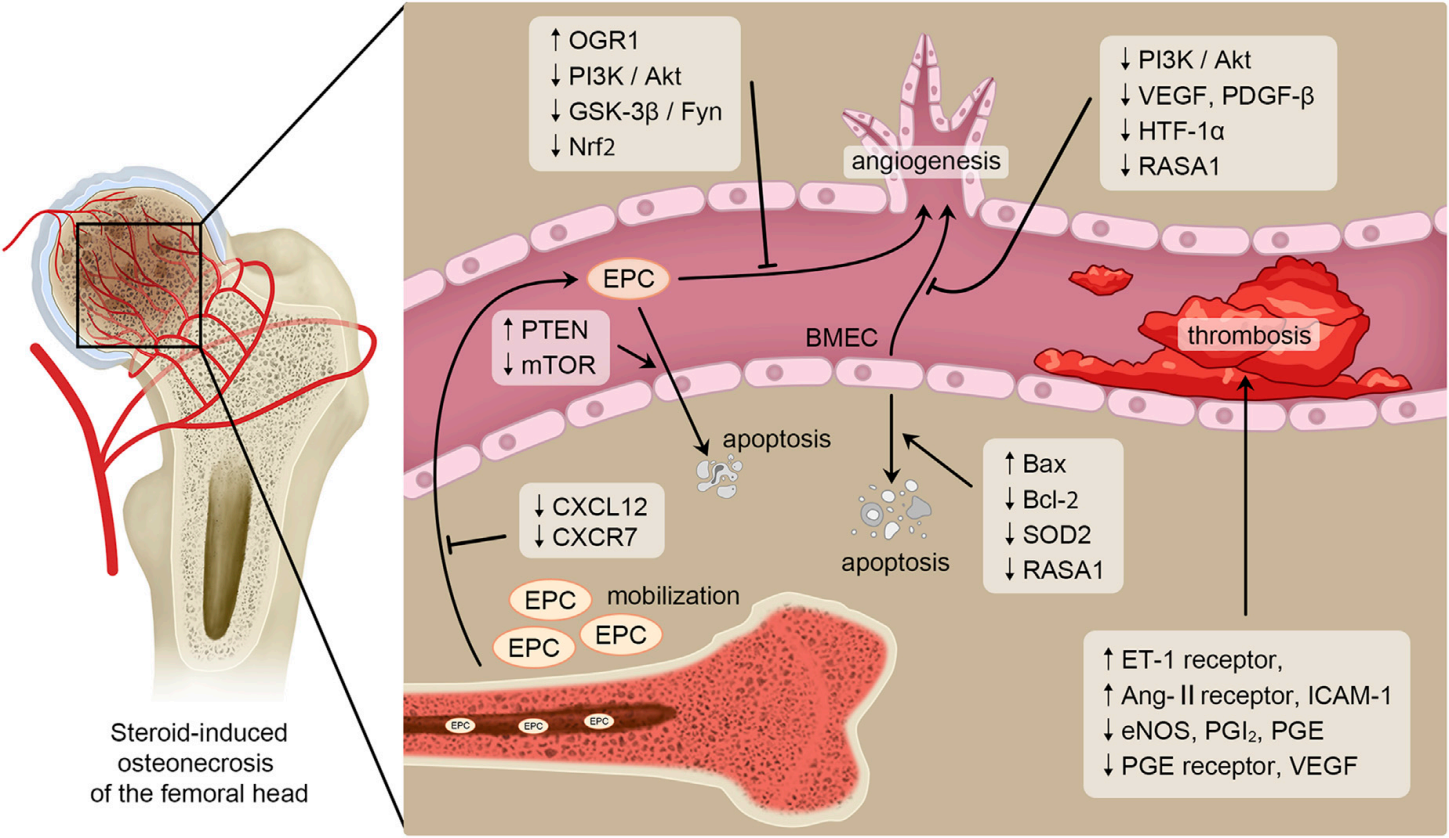
Pathogenesis in SONFH related to bone endothelial cells. Blood vessels play a critical role in the occurrence and progression of SONFH. And bone endothelial cells are essential for maintaining vascular homeostasis and angiogenesis. Therefore, bone endothelial cells are key regulatory factors in the pathogenesis of SONFH. SONFH is affected by GCs regulating mobilization, angiogenesis, apoptosis and thrombosis of bone endothelial cells through several signaling pathways or cytokines such as PI3K/Akt, GSK-3β/Fyn, Bcl-2 and Bax.

SONFH is a disabling joint disease without effective drug treatment so far. Severe advanced SONFH can only be treated with THA, which may be accompanied by a series of side effects, including dislocation, periprosthetic fracture, infection and prosthesis loosening especially for young, active population. Impaired blood vessels is a key factor in many of the proposed pathogenesis of SONFH. Bone endothelial cells, including BMECs and their precursors, EPCs, both play a critical role in maintaining the normal structure and function of blood vessels. Impaired angiogenesis, abnormal apoptosis, thrombosis and fat embolism caused by the dysfunction of bone endothelial cells are involved in the occurrence and progression of SONFH (Figure 1). Therefore, ICA, pravastatin, and VO-OHpic are candidate reagents for the prevention and treatment of SONFH by promoting angiogenesis and inhibiting apoptosis and vascular embolization (Table 1). However, these reagents are still in the preclinical stage and are not yet sufficient for widespread clinical use. In addition, bone tissue engineering such as bone endothelial cells and BMSCs co-transplantation is one of the most promising strategies for treating SONFH. The optimal ratio between cultured cells of co-grafts and scaffolds with excellent biocompatibility need further investigations.

**TABLE 1:** Candidate reagents targeting bone endothelial cells to treat SONFH.

Candidate reagents	Experimental models	Effects	References
ICA	BMECs isolated from LPS and MPS treated rats	Decreased miR-335 to up-regulate the expression of eNOS, SOD2, RASA1	Yue et al. (2021)
	BMECs isolated from MPS treated rats	Decreased the rate of empty lacunae and increased blood vessels and the angiogenic biomarker CD31	Yu et al. (2019)
	BMECs isolated from patients undergoing THA with femoral neck fractures	Promoted angiogenesis by up-regulating the expression of CD31, vWF, PDGF-B in BMECs and activating Akt and reduced the apoptosis of BMECs by up-regulating Bax and down-regulating the expression of Bcl-2	Yu et al. (2019)
Pravastatin	EPCs isolated from Dex treated rats	Activated AMPK mediated by LKB1, thereby inhibiting the mTOR signaling pathway, recovering autophagy of EPCs and protecting them from Dex-induced apoptosis	Liao et al. (2018)
VO-OHpic	EPCs isolated from rats and treated with MPS	Promoted angiogenesis and suppressed apoptosis through inhibiting mitochondrial pathway and activating Nrf2	Yao et al. (2020)

Abbreviations: ICA, icariin; BMECs, bone microvascular endothelial cells; LPS, lipopolysaccharide; MPS, methylprednisolone; eNOS, endothelial nitric oxide synthase; SOD2, superoxide dismutase 2; RASA1, Ras p21 protein activator 1; THA, total hip arthroplasty; vWF, von Willebrand factor; PDGF-B, platelet-derived growth factor-B; Bax, Bcl-2, associated X; Bcl-2, B cell lymphoma-2; EPCs, endothelial progenitor cells; Dex, dexamethasone; AMPK, AMP-activated protein kinase; LKB1, liver kinase B1; mTOR, mammalian target of rapamycin; Nrf2, nuclear factor erythroid 2-related factor 2.
